# Regional Patterns in Retinal Microvascular Network Geometry in Health and Disease

**DOI:** 10.1038/s41598-019-52659-8

**Published:** 2019-11-08

**Authors:** Natasa Popovic, Stela Vujosevic, Tomo Popovic

**Affiliations:** 1 Faculty of Medicine, University of Montenegro, Kruševac bb, Podgorica, Montenegro; 2 Eye Clinic, University Hospital “Maggiore della Caritá”, Novara, Italy; 3 Faculty for Information Systems and Technologies, University of Donja Gorica, Oktoih 1, Podgorica, Montenegro

**Keywords:** Computational biology and bioinformatics, Physiology, Biomarkers, Medical research, Risk factors

## Abstract

The study explores the regional differences in microvascular geometry between the optic disc (O) and the macular area (M) in health and disease. Skeletonized manually segmented vascular networks from 15 healthy, 15 retinas with diabetic retinopathy (DR), and 15 retinas with glaucoma from publicly available High-Resolution Fundus (HRF) image database were used. When visualized by a digital fundus camera, O has a substantial proportion of small arteries and larger arterioles, while M contains smaller arterioles at the periphery and avascular zone in the center. We hypothesized that in pathological conditions the vascular network remodelling patterns in these two regions may be different. The analysis of box-counting fractal dimension (Db), lacunarity (Λ), and microvascular density showed that in healthy retinas, Λ and vessel density were lower in the M compared to the O, while the Db did not change. In retinas with DR, the Db was the lowest in the M, which was different from all other groups. The vessel density followed this trend. Lacunarity was the highest in the O of DR group compared to all other groups. The results show that in DR various regions of retinal microvascular network remodel in a different manner and to different extent.

## Introduction

There is growing scientific evidence that the examination of retinal microvasculature could be used as a non-invasive and inexpensive method for evaluating the condition of systemic microvascular circulation in health and disease^1^. The circulatory tree has a fractal structure and the first use of fractal parametrization to measure the complexity and self-similarity of a retinal microvascular network in order to describe its geometry dates back to 1989^2^. However, although fractal structure of the retinal vasculature has been extensively studied since, the results have had limited application in real life due to its low specificity in disease characterization. For example, decreased complexity of microvascular retinal network measured by fractal dimension is associated with the presence of cardiovascular risk factors such as arterial hypertension (HTN), diabetes mellitus (DM) and old age^1^. At the same time decreased fractal dimension of retinal microvasculature is associated with some eye - specific diseases such as cataract and high myopia^3^.

Although the remodelling of retinal microvascular network in pathologic conditions affects retina globally, the differences in the extent and type of remodelling among various segments of retinal microvascular network may exist^4,5^. These differences in remodelling may stem from the distinct function and histological structure among microvessels of various size^6^. It has been reported that the diameters of blood vessels generally decrease as their number of branching generations increases^7,8^. The blood vessels belonging to the retinal arterial circulation that can be detected by a fundus camera originate at the optic disc area, branch and all achieve arteriolar status at about 0.5 optic disc diameters from the optic disc margin^1,9^. While small arteries and arterioles contain smooth muscle cells in the vessel wall and are able to control vascular resistance and blood flow, the capillaries contain only one layer of endothelial cells, have diameter around 7 μm, and mainly participate in nutrient exchange^6,10^. In hypertension, one of the hallmark signs of hypertensive remodelling of small resistance arteries is increase of media-to-lumen ratio through the processes known as hypertrophic and eutrophic remodelling. On the other hand, the capillaries, arterioles, and venules of the microvascular network in essential hypertension remodel through rarefaction^6^. Therefore, we could argue that although rarefaction and decreased complexity of microvasculature are global processes affecting retinal microvasculature in hypertension^1^, they may not be present to the same extent in all regions of retina, especially in cases where the average vessel diameter is different.

Inspired by these observations, we hypothesized that various regions of microvascular network within the retina may remodel to a different extent and sometimes in a different manner, resulting in remodelling patterns that are unique to the type of causative pathological process. To test this hypothesis, we compare microvascular geometry of the two regions of interest (ROI) in the retina that functionally and structurally represent two distinct regions, the optic disc area (O) and the macular region (M). The optic disc area is devoid of photoreceptors. Contrary to this, macular region is known to be in charge of acute, photopic, high resolution color vision^11^. When visualized by a digital fundus camera, retinal vasculature in optic disc area has a substantial proportion of small arteries and larger arterioles^1^. On the other hand, the macular area visualized by a digital fundus camera is characterized by smaller arterioles at the periphery (peri - foveal area), while the very center of this area is avascular (foveal avascular zone – FAZ)^12^.

In our study, we used a publicly available High-Resolution Fundus (HRF) image database containing 45 high-resolution color fundus photographs, each associated with manually segmented vascular network^13,14^. This database contains a group of images of 15 healthy retinas, 15 fundus images with diabetic retinopathy (DR), and 15 images of retinas associated with the diagnosis of glaucoma. Microvascular changes in the retina during DR represent the manifestation of a systemic disease, while in a considerable number of patients microvascular changes in the retina caused by glaucoma represent the manifestation of an eye-specific disease^15,16^. In our study we first described the patterns of microvascular geometry of the two functionally and anatomically distinct ROI in healthy retina by using box counting fractal dimension, lacunarity, microvascular density parameters. Following that, we examined how the patterns of microvascular geometry change due to remodelling caused by pathological processes such as glaucoma and DR at the global level - in the whole retina, and at the local level - in these two specific ROI.

## Results

### Ophthalmologic characterization of images

It has been shown that the morphology of the microvascular network is strongly affected by the stage of DR. For example, some of the morphological features typically found in DR such as vascular pruning and neovascularization, can affect vessel density in opposing manners, and they appear at different stages of DR. Neovascularization is a hallmark feature of advanced i.e. proliferative DR, while vascular pruning can be observed even at earlier stages of DR^17^. In support of this observation, Parsons-Wingerter *et al*. found that during the progression of DR retinal vessel density oscillates: it initially increases when mild progresses to moderate DR, then it decreases in severe non-proliferative DR, and finally in the severe proliferative stage it increases again. This change was most pronounced in smaller blood vessels belonging to the 6^th^ or even higher generations of vessel branching^18^. Therefore, the images from HRF database were graded by an experienced ophthalmologist in order to determine the stage of DR, presence of other pathological changes and treatment - related artefacts in the retina. The results in Table 1 show that the majority retinas with DR display signs of severe non-proliferative and severe proliferative DR.

**Table 1:** Retinal fundus photograph grading results.

ID	No apparent retinopathy	NPDR			PDR	Laser	Retinal lesions
		mild	moderate	severe			
01DR			X			X	MAs, haemorrhages
01G	X						
01H	X						
02DR					X	X	MAs, haemorrhages, neovascularization
02G	X						
02H	X						
03DR				X		heavy	
03G	X						
03H	X						
04DR				X		heavy	maculopathy
04G	X						
04H	X						
05DR	X						
05G	X						
05H	X						
06DR					X		maculopathy
06G	X						
06H	X						
07DR				X			MAs, haemorrhages, IRMAs
07G	X						
07H	X						
08DR				X		X	severe maculopathy
08G	X						
08H	X						
09DR					X	X	venous beading, MAs, haemorrhages, IRMAs, neovascularization
09G	X						
09H	X						
10DR	X						
10G	X						
10H	X						
11DR			X				MAs, maculopathy
11G	X						
11H	X						
12DR					X	X	neovascularization
12G	X						
12H	X						
13DR				X		X	MAs, haemorrhages, IrMAs
13G							
13H							
14DR					X	X	neovascularization
14G	X						
14H	X						
15DR							CRVO
15G	X						
15H	X						

DR: diabetic retinopathy, G: glaucoma, H: healthy, NPDR: non-proliferative diabetic retinopathy, PDR: proliferative diabetic retinopathy, MAs: microaneurysms, IRMAs: intraretinal microvascular abnormalities, CRVO: central retinal vein occlusion.

### Estimation of the vessel diameter in the retinal vascular tree

Although vascular remodelling affects retinal microvasculature globally during the disease^1^, some studies found that remodelling may not be present to the same extent in all regions of retina, especially in cases where the average vessel diameter is different^18^. To be able to test this in the present study, we estimated the size and the branching generation of the blood vessels detected by the fundus camera in the two ROIs. The estimated average size of the largest vessels belonging to both, arterial and venous vascular trees, crossing the optic disc margin was 98.72 μm (range from 88 to 114 μm). After approximately 5 generations of branching, the average size of the smallest blood vessels that could be detected by the fundus camera in the area of the macula was 21 μm (range from 19 μm to 28 μm). Therefore, the blood vessels in the optic disc ROI belong to the category of small arteries and veins, as well as venules and arterioles that generally originate from low number of branching generations. On the other hand, the blood vessels detected in the area of macula mostly belong to the category of smaller venules and arterioles belonging to the higher number (up to the 5^th^ generation) of branching generations (Supplementary Fig. 1).

### Characterization of microvascular network in healthy retinas

**Figure 1:**
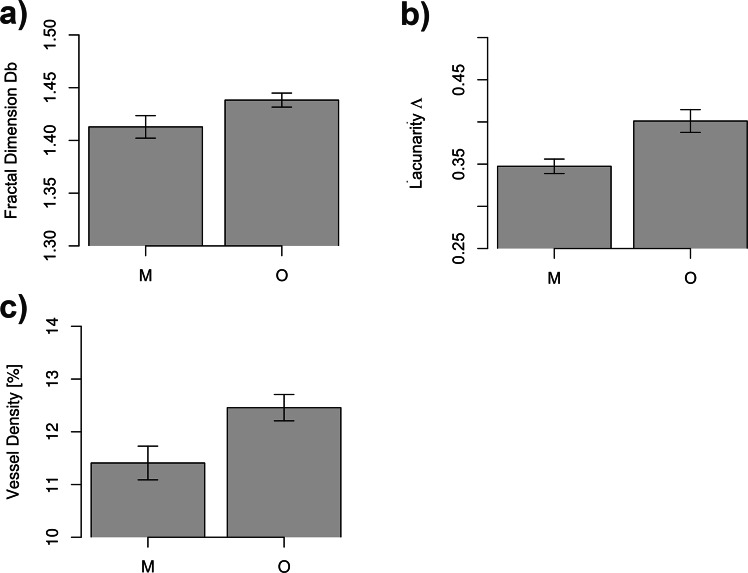
Regional differences in microvascular network geometry in healthy retina. Macular (M) vs. optic disc region (O): (**a**) mean fractal dimension (Db ± SD): 1.41 ± 0.04 for M vs. 1.44 ± 0.03 for O, p = 0.05; (**b**) mean lacunarity (Λ ± SD): 0.35 ± 0.03 for M vs. 0.40 ± 0.05 for O, p = 0.002; (**c**) mean skeletonized vessel density (Vessel Density [%] ± SD): 11.41 ± 1.24 for M vs. 12.46 ± 0.97 for O, p = 0.015. All analyses were performed on skeletonized images. P- values < 0.05 are considered significant.

In healthy retinas, the analysis of microvascular network geometry by fractal dimension, lacunarity and vessel density showed that lacunarity and vessel density are lower in the region of macula compared to optic disc region, while the fractal dimension did not change (Fig. 1).

### Global patterns of the microvascular network geometry in pathological states

At the global level, lacunarity is increased in DR compared to the healthy group. However, microvascular complexity and vessel density are not different among the groups (Table 2).

**Table 2:** Parameters of vascular network morphology of the whole retina in pathological states.

Diagnosis	DR	G	H	p-value
Box counting dimension (Db) mean ± SD	1.57 ± 0.03	1.58 ± 0.02	1.57 ± 0.02	0.23
Lacunarity (Λ) mean ± SD	0.37 ± 0.06^a^	0.34 ± 0.03^ab^	0.33 ± 0.02^b^	0.03*
Skeletonized vessel density mean[%] ± SD	26.11 ± 2.90	27.39 ± 2.26	26.9 ± 2.06	0.359

All analyses were performed on skeletonized images. *P- values < 0.05 are considered significant. Groups labelled with different letters are statistically different.

### Regional patterns of the microvascular network geometry in pathological states

**Figure 2:**
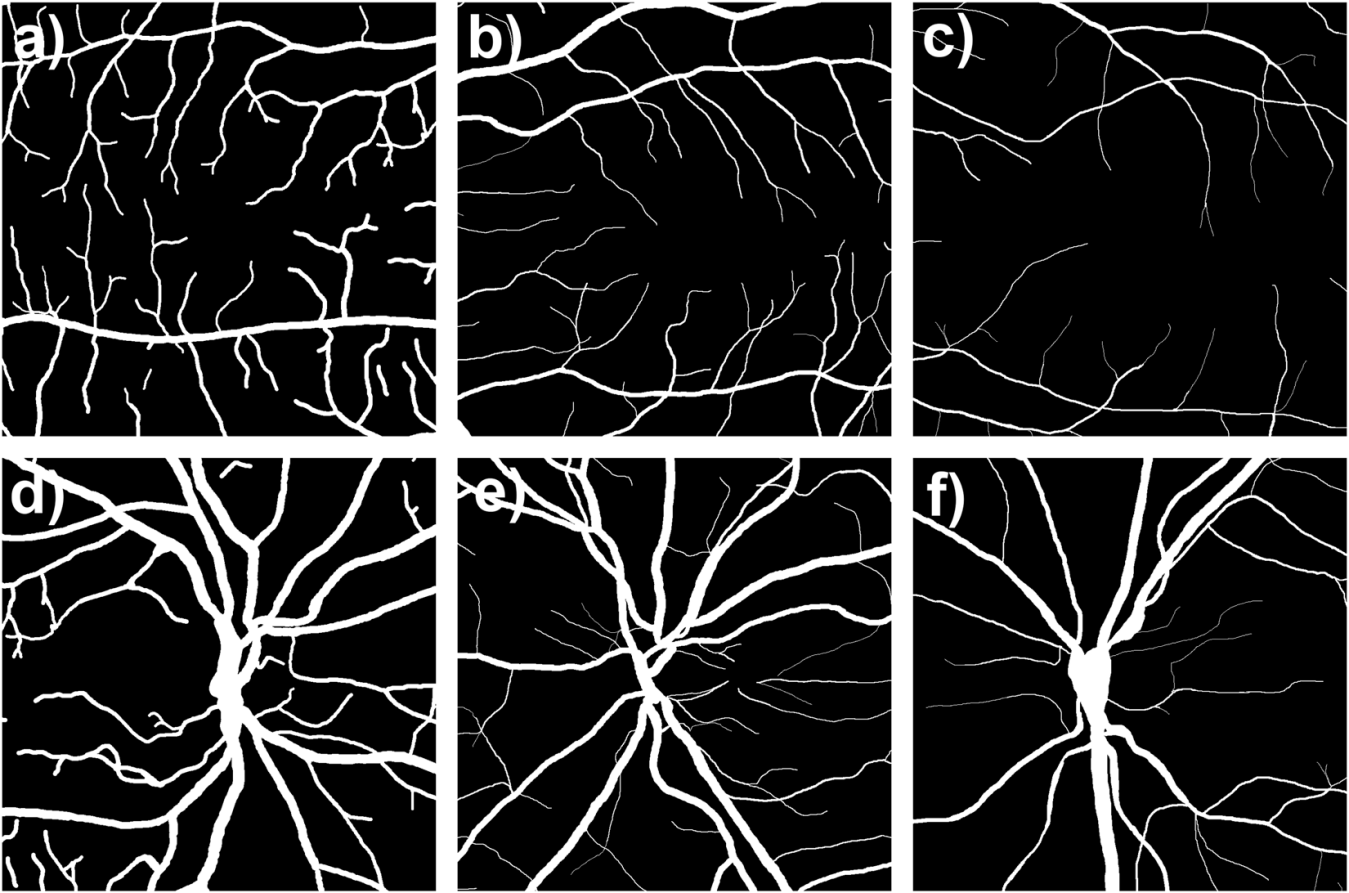
Visual comparison of the manually segmented images of the two ROIs. MACULAR region: (**a**) healthy retina, (**b**) glaucoma, (**c**) DR; OPTIC DISC region: (**d**) healthy retina, (**e**) glaucoma, (**f**) DR. The blood vessel space filling property in the image appears to be the lowest in macular region of retinas with DR.

**Figure 3:**
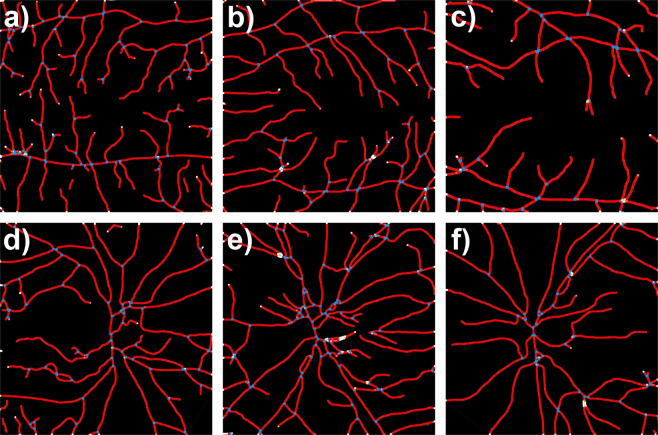
Visual comparison of the skeletonized microvascular images of the two ROIs. Skeletonized vessel density [%] and microvascular complexity appear to be the lowest in the macular region of retina with DR. MACULAR region: (**a)** healthy retina, (**b**) glaucoma, (**c**) DR; OPTIC DISC region: (**d**) healthy retina, (**e**) glaucoma, (**f**) DR.

Visual comparison of the two ROI suggests that the space filling property of the vascular network is generally lower in the macular region when compared to the optic disc area. This is especially pronounced in the macular region of retinas with DR (Fig. 2). These differences in space filling properties may stem from the fact that the macular region includes mostly smaller caliber, while optic disc region contains mostly larger caliber microvessels. In order to eliminate this space filling effect, we performed skeletonization of all manually segmented images before we proceeded with further analysis. Visual comparison of the skeletonized images suggests that vessel density and complexity may be the lowest in the macular region of the retinas with DR as well (Fig. 3).

**Figure 4:**
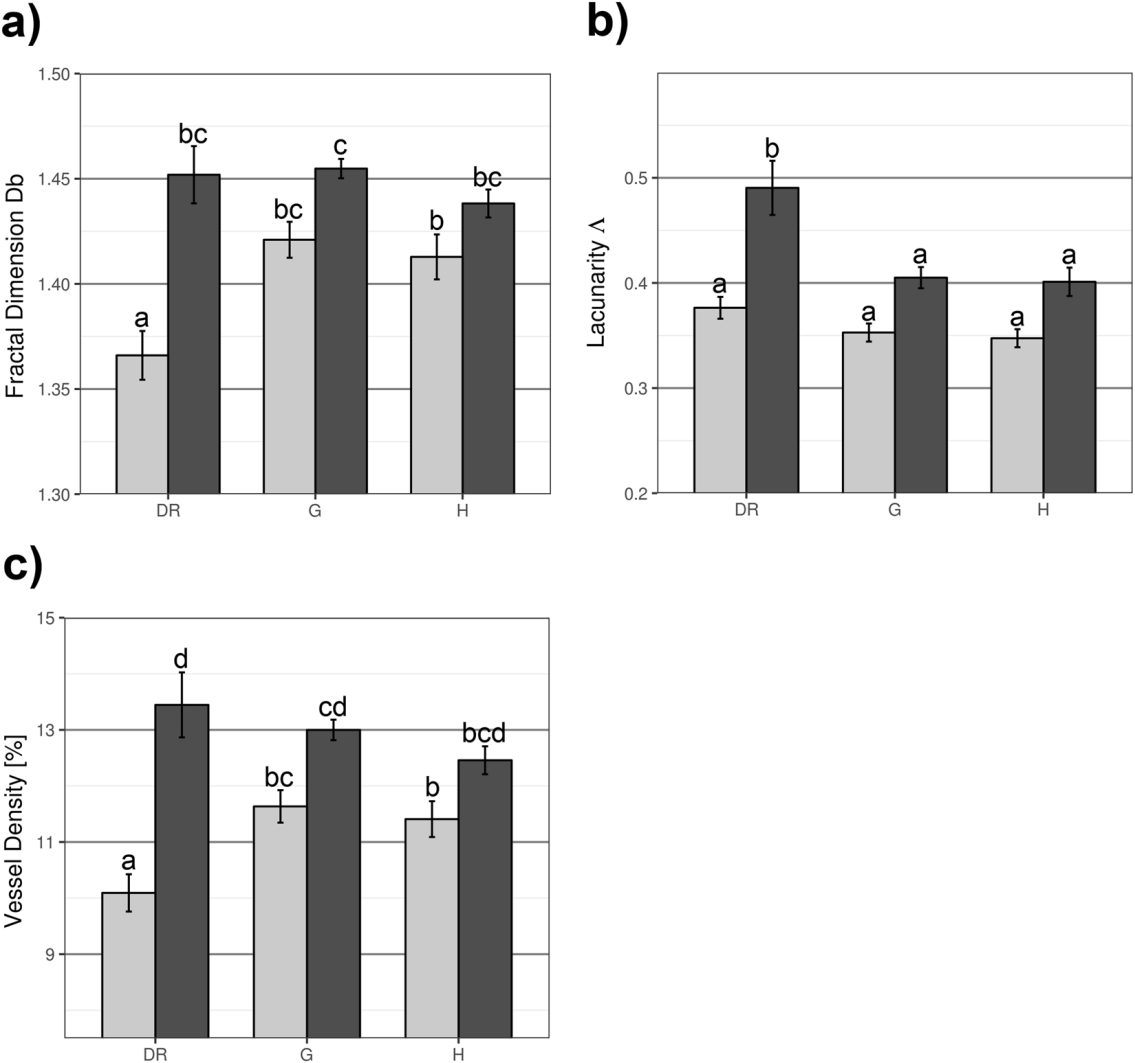
Regional patterns of microvascular network remodelling in DR and glaucoma. (**a**) Box counting dimension (Db), (**b**) Mean lacunarity (Λ), (**c**) Skeletonized vessel density [%]. Light bars - macular region (M), Dark bars - optic disc region (O). Bars labelled with different letters are statistically different.

Box counting analysis confirmed the results of the visual inspection and showed that this decrease in complexity was the most pronounced in DR group in the macular region (Fig. 4a). The vessel density parameter followed the same trends (Fig. 4c). In addition, in the optic disc area, lacunarity in DR was significantly higher compared to all other groups of images (Fig. 4b). Taken together the results show that DR group of images displayed a distinct pattern of microvascular geometry when described by the 3 parameters together (Table 3).

**Table 3:** Summary of the results showing regional patterns in vascular network geometry in glaucoma and DR.

Region	Macular			Optic disc			p-value
Diagnosis	DR	G	H	DR	G	H	
Box counting dimension (Db) mean ± SD	1.37 ± 0.04	1.42 ± 0.03	1.41 ± 0.04	1.45 ± 0.05	1.45 ± 0.02	1.44 ± 0.03	0.005*
Lacunarity (Λ) mean ± SD	0.38 ± 0.04	0.35 ± 0.03	0.35 ± 0.03	0.49 ± 0.10	0.40 ± 0.04	0.40 ± 0.05	0.049*
Skeletonized vessel density[%] ± SD	10.09 ± 1.29	11.63 ± 1.12	11.41 ± 1.24	13.45 ± 2.24	13 ± 0.71	12.46 ± 0.97	0.002*

*P- values < 0.05 are considered significant.

## Discussion

This study shows that in healthy retina visualized by a digital fundus camera, the geometry of microvascular network varies significantly depending on the ROI. In addition, we documented the specific changes of these patterns that occur in the advanced stages of DR.

Other researchers that conducted a global analysis of microvascular complexity found that retinal microvascular network complexity in glaucoma decreases on the global level^19^. For DR, the reports have been less conclusive so far. While some studies showed decrease in vascular network complexity and increase in lacunarity^20,21^, others showed increase in complexity^22^. The results of the global analysis we present here failed to demonstrate a significant change in microvascular network complexity in either of these two pathologic processes. In addition, our global analysis showed that lacunarity in retinas with DR was increased compared to the healthy group, but was not different from the group with glaucoma. The reasons for these disagreements with other published literature may be the lack of statistical power due to the small sample size used in our study, or possibly the use of different imaging techniques.

This study compares two regions of the retina characterized by significant innate functional and morphological differences. Other researchers analyzed retinal vascular network geometry in a region-specific manner as well. For example, Avakian *et al*. and Ventura LolaCosta *et al*. divided retina in a geometric manner to 9 equal squares in patients with DR^4,23^. Aliahmad *et al*. used retinal fundus images of the patients who suffered stroke and analyzed doughnut-shaped ROIs that were centred around the optic disc^24^. Cabrera DeBuc *et al*. also defined ROIs in a disc-centric fashion to study the relationship between retinal network complexity and neurodegenerative changes in patients with cognitive impairment^9^. Zhang *et al*. studied doughnut-shaped parafoveal and paramacular areas in the retina of patients with mild cognitive impairment and early Alzheimer’s dementia^25^. However, most of these studies were aimed to find a single most important ROI in the retina in order to detect certain disease, predict the rate of its progression, screen for the complications and/or measure the effects of therapy.

The studies that focus on examining differences in vascular network morphology among the various regions of retina in health and disease are relatively rare in the currently available literature. One such study^4^ examined microvascular morphology in patients with mild to moderate DR and it showed that fractal dimension decreased in macular region, but this trend was not observed in paramacular regions, or when the retina was analyzed as a whole. This observation is in agreement with our results. In the present study the global fractal analysis did not show differences in microvascular network complexity among the groups, while the analysis focused on the two ROI showed that complexity decreases in the macular region of retinas with DR (Fig. 4a). In addition, although the location-specific analysis showed that vessel density decreases specifically in macular region in DR (Fig. 4c), the decrease in vessel density was not observed in the global analysis. Global lacunarity analysis showed that lacunarity increases in DR when compared to healthy retinas, but this increase was not different from retinas with glaucoma. However, if lacunarity was compared locally with the focus on the two ROIs, it was specifically increased in the optic disc area of retinas with DR which was different from all other groups. Taken together these results suggest that inclusion of the whole retina, or the larger areas of retina in the analysis sometimes can cause averaging of the parameter that is being examined. This is an important observation because various regulators of vasculogenesis such as FGF, and TGFβ could affect vascular patterning at the local level, through processes related to the specific branching generations of the vascular tree^26,27^. Here, we present the results of location-specific and branching generation-specific analysis of retinal microvascular geometry that are in agreement with these observations.

The results of our location-specific analysis demonstrated that in DR fractal dimension and vascular density decrease mostly due to changes in the microvasculature in the macular area. Microvascular paucity and the increase in size of FAZ, the well-known microvascular changes associated with DR^17,28^, may be the reason why decrease in blood vessel density and fractal dimension were the most pronounced in macular ROI. It is important to emphasize that the analysis presented here encompasses only the segment of microvascular network starting from the central retinal artery and vein and their arteriolar and venular branches up to approximately 5 generations of branching. This limitation is a result of technical capabilities of the fundus camera used to capture the images. Contrary to these observations, several studies using OCT- angiography have shown decreased vessel density at the level of the radial capillary plexus located in the most superficial retinal layers, in the proximity of the optic nerve. These changes are very precociously present in patients with diabetes mellitus, even before clinical signs of DR are present and before the microvascular changes in the macular region occur^29^. However, in the present study, the vascular complexity and density in the macular region was affected, while the optic disc region was not affected. This may be caused by the fact that in the present study most DR images represent more advanced stages of DR. Also, only large retinal vessels up to the 5^th^ branching generation, visible on fundus color photographs were evaluated in the present study, whereas in the previous studies OCT - angiography was used, which evaluated small retinal capillary and precapillary vessels, while larger retinal vessels were excluded from the analyses. The findings of the OCT- based studies support our hypothesis that the same pathological process can cause various regions of microvascular network within retina to remodel to a different extent and sometimes in a different manner.

The present study does not address the differences between arteriolar and venular geometry, or type and stage of diabetes mellitus and glaucoma. Most of the retinal fundus images belonging to the patients with the advanced DR have laser treatment artefacts, haemorrhages, microaneurysms, and intraretinal microvascular abnormalities, which could have influenced the results (Table 1). More importantly, the retinal fundus images are not accompanied with any demographic, or any other relevant clinical data that would allow us to accurately describe causative or temporal relationships between the microvascular network geometry and a certain disease, it merely shows their associations. Therefore the results related to disease-specific patterns of microvascular remodelling we present here should be interpreted with caution. Nevertheless, our study explores a new approach in the analysis of retinal microvascular network geometry, and demonstrates that studying regional differences in this geometry can be more informative than selecting one region of interest, or trying to characterize the geometry of the whole retinal vascular tree by one average number. In order to increase specificity of this type of analysis, it will be necessary to examine more parameters characterizing microvascular network geometry at the same time. For example, the microvascular network of retina displays multifractal geometry^30–32^. Although we did not address this in the present study, it is something we definitely intend to focus on in the future large prospective studies.

Taken together, the study presented here shows that looking at the regional differences in microvascular network morphology in certain disease states might be a more powerful approach in studying that disease than characterizing the microvascular network of retina as a whole. These ROI should not be chosen randomly, instead, they should be selected according to their unique structure and function. Our findings reinforce the fact that the use of even a low-cost non-midriatic portable fundus camera, capable of capturing only limited regions of retina at a time, could serve as a useful tool to study regional differences in vascular network geometry. In the future, large prospective studies that account for all the relevant clinical data could help us to increase our knowledge on hallmark patterns of regional microvascular remodelling and thereby improve detection various systemic and ocular diseases.

## Methods

### Retinal images

The study uses a publicly available High - Resolution Fundus (HRF) image database containing 45 raw color retinal fundus images, with a resolution of 3504 × 2336 pixels, captured with a Canon CR-1 digital fundus camera with a 45° field of view^13,14,33^. The database contains 3 groups of images: 15 images of healthy retina, 15 images of retina with signs of DR (one image is with central retinal vein occlusion) and 15 with signs of glaucoma. Each image has a corresponding gold standard vessel segmentation binary image, and data on optic disc localization^13,14^. The expert ophthalmologist with extensive experience in grading of retinal images (S.V.), also determined the location of macula in each image (Supplementary Table 1), and graded the retinal fundus photographs (Table 1). Demographic and other clinical data associated with the study participants, as well as the settings under which the images were captured were not available.

### Regions of interest

Manually segmented retinal images were cropped by using a custom Python script to yield 2 square ROI, one centred on the optic disc, and the other centred on the macular region. The size of each ROI was arbitrarily selected to be 1000 × 1000 pixels. Subsequently, the same program was used to produce similar circular ROI, each 1000 pixels in diameter.

Both, square and circular ROIs, have been used in the past by researchers examining regional changes in retinal microvascular geometry^23,24^. While the use of circular ROI is intuitively reasonable because they follow intrinsic shape of the eye, the square shape has a technical advantage because the subsequent box counting fractal analysis, with settings we used in the study, divides image into smaller square boxes, which is a straightforward process if the ROI has a square or rectangular shape. However, the box counting fractal analysis of the circular images introduces spurious black foreground areas at the corners to convert them to the square shape.

After we skeletonized both sets of images and performed box counting and lacunarity analysis, the results showed that in both sets of images groups display the same trends (Supplementary Fig. 2). In the rectangular ROIs, the absolute values for fractal dimension (Db) are generally higher and mean lacunarity (Λ) values are generally lower when compared to the circular ROIs. The p-value for Db is significant for both the rectangular and circular ROI, with the Db for the macular region of retinas with DR is different from all other groups in both analyses. The p-value for Λ is borderline significant for the rectangular ROIs, with the optic disc region of DR group being different from all other groups, while for the circular ROI p-value is not significant (Supplementary Table 2). The cause for these inconsistencies between rectangular and circular ROIs may be the presence of the black foreground in the corners of the images with the circular ROI as they are processed by the ImageJ algorithm as an empty space. Therefore, we chose to use rectangular ROI for all further analyses.

### Estimation of blood vessel diameters

To confirm the caliber of blood vessels that are typically visualized in the two ROIs by the fundus camera, we estimated the average blood vessel diameter in μm. We consider this question important to the original aim of the study because different types of the blood vessels remodel in a different manner, which may be the ultimate cause for different microvascular network patterns in the two ROI in the disease states^6^. To estimate the blood vessel diameters, we used the approach similar to the one applied by Knudtson *et al*.^7^. The known average horizontal and vertical optic disc dimensions in μm in the healthy eyes^34^ were compared to the corresponding average optic disc dimensions in pixels of the 15 images of healthy retina in the database used in our study. Based on this, the estimated size of one pixel in the raw images was 4.86 μm. Then, blood vessel diameter was measured on 5 randomly selected raw images from each group viewed in gray scale mode by using Gnu Image Manipulation program (GIMP). In each image, the external diameters of the five largest blood vessels crossing the optic disc margin, and 5 smallest blood vessels detected by the fundus camera in the macular region were measured. This approach allowed us only to estimate the diameters of the blood vessels. However, this was sufficient to confirm that the vessels in the two ROIs mostly belong to the category of arterioles and venules, and that due to technical limitations of fundus camera no capillaries were represented in the segmented images. The method we used was not precise enough for comparisons of the vessel diameters between the two ROIs and/or among the experimental groups. The method did not account for the possible variations in magnification caused by refractive errors of the lens, or variations in the axial length of the eye. Finally, the margins of the vessel wall were better defined in larger vessels, which may have caused more frequent measurement errors when measuring calibers of small vessels, which is a technical issue noted by other researchers as well^7^.

### Estimation of the number of branching generations

To estimate the number of branching generations in each ROI, we used a randomly selected manually segmented binarized image of microvascular network of the whole healthy retina, and applied the rules based on the gradual decrease in vessel diameter associated with branching described by Vickerman *et al*.^8^. In short, the vessels in the area of the optic disc with the largest diameter belong to the 1^st^ generation of branching. In most cases, these vessels branch asymmetrically to produce a daughter vessel that has a much smaller diameter than the parent vessel. In that case, the parent vessel would keep its branching generation designation, while the daughter vessel would belong to the next branching generation. In some cases, the parent vessel bifurcates into two branches with relatively equal diameters. In this case both daughter vessels would belong to the next branching generation (Supplementary Fig. 1).

### Box counting and lacunarity analysis

The manually segmented binary images of vascular trees were first skeletonized in ImageJ software version 1.52a by using Skeletonize (2D/3D) plugin^35^. Subsequently, the box counting and lacunarity analysis were performed in ImageJ by using the FracLac plugin^35^ according to formulas described previously^21^. The box counting analysis algorithm was applied with the following settings: a) grid design: number of origins = 12, rotation and random number generator options = off, sampling element shape = square, use the seed option is off; b) scaling method was the “default linear sampling sizes” that uses a non-overlapping square sampling element with the range of sizes from a minimum size of 0 pixels to a maximum size of 45%. A higher box counting dimension (Db) means that the microvascular network has a more complex pattern. Heterogeneity of the images was expressed as the lacunarity for the image where one number for each image represents heterogeneity over all grid orientations. This value is calculated by default for all fixed grid scans and is found in the FracLac results file. Higher values for lacunarity dimension (Λ) means that the microvascular network has greater inhomogeneity or gappiness.

### Vessel density

In order to determine the vessel density in each ROI, the manually segmented ROIs were first skeletonized by using ImageJ, then analyzed by using Angiotool software that was downloaded separately^36,37^. The vessel density was calculated as a percent vessel area of the skeletonized vascular trees. Skeletonization eliminated bias that would be caused by inherent variations in the vessel diameter.

### Statistical analysis

Statistical analysis was performed by using the statistical analysis software R. Independent sample two-tailed Student’s T-test was used for data analysis to characterize the microvascular network geometry in the healthy retinas. One-way ANOVA with Tukey post-hoc test was used to analyze the global patterns of the microvascular network geometry in health and disease. Two-way ANOVA with Tukey post-hoc test was used to study the regional patterns of the microvascular network geometry in health and disease. P values < 0.05 were considered significant.

## Supplementary information

Supplementary information

## Data Availability

The data sets generated and analyzed in the presented study are available from the corresponding author on reasonable request.
