# Supplementary material for: Regional Patterns in Retinal Microvascular Network Geometry in Health and Disease
Source: Sci Rep. 2019 Nov 8;9:16340. doi: 10.1038/s41598-019-52659-8 (PMC6841983; doi:10.1038/s41598-019-52659-8)
Supplement: Supplementary file 1 — Supplementary information [file 41598_2019_52659_MOESM1_ESM.docx]

**SUPPLEMENTARY INFORMATION**

**Manuscript title:**

Regional Patterns in Retinal Microvascular Network Geometry in Health and Disease

**Authors:**

Natasa Popovic, MD, PhD^1*^, Stela Vujosevic, MD, PhD^2^, Tomo Popovic, PhD^3^

^1^Faculty of Medicine, University of Montenegro, Kruševac bb, Podgorica, Montenegro

^2^Eye Clinic, University Hospital “Maggiore della Caritá”, Novara, Italy

^3^Faculty for Information Systems and Technologies, University of Donja Gorica, Oktoih 1, Podgorica, Montenegro

| ID | Macula center X | Macula center Y |
| --- | --- | --- |
| 01DR | 1740 | 1144 |
| 01G | 1856 | 1216 |
| 01H | 1824 | 1184 |
| 02DR | 1840 | 1184 |
| 02G | 1676 | 1184 |
| 02H | 1848 | 1252 |
| 03DR | 1796 | 1284 |
| 03G | 1764 | 1152 |
| 03H | 1848 | 1236 |
| 04DR | 1864 | 1360 |
| 04G | 1812 | 1264 |
| 04H | 1812 | 1240 |
| 05DR | 1812 | 1264 |
| 05G | 1828 | 1260 |
| 05H | 1812 | 1236 |
| 06DR | 1829 | 1256 |
| 06G | 1828 | 1260 |
| 06H | 1808 | 1236 |
| 07DR | 1808 | 1244 |
| 07G | 1832 | 1304 |
| 07H | 1816 | 1268 |
| 08DR | 1764 | 1256 |
| 08G | 1776 | 1264 |
| 08H | 1772 | 1160 |
| 09DR | 1836 | 1288 |
| 09G | 1768 | 1248 |
| 09H | 1804 | 1244 |
| 10DR | 1848 | 1236 |
| 10G | 1780 | 1260 |
| 10H | 1808 | 1228 |
| 11DR | 1888 | 1256 |
| 11G | 1844 | 1268 |
| 11H | 1956 | 1240 |
| 12DR | 1760 | 1140 |
| 12G | 1840 | 1252 |
| 12H | 1904 | 1156 |
| 13DR | 1760 | 1128 |
| 13G | 1864 | 1228 |
| 13H | 1784 | 1228 |
| 14DR | 1796 | 1236 |
| 14G | 1856 | 1276 |
| 14H | 1756 | 1204 |
| 15DR | 1800 | 1236 |
| 15G | 1788 | 1232 |
| 15H | 1788 | 1216 |

**Supplementary table 1:** Location of macula center in each raw image from the High – Resolution Fundus (HRF) image database^13,14^.

| Rectangular skeletonized ROI | | | | | | | |
| --- | --- | --- | --- | --- | --- | --- | --- |
| Region | Macular | | | Optic disc | | |  |
| Diagnosis | DR | G | H | DR | G | H | p-value |
| Box counting  dimension  (Db)  mean±SD | 1.37±0.04 | 1.42±0.03 | 1.41±0.04 | 1.45±0.05 | 1.45±0.02 | 1.44±0.03 | 0.005* |
| Lacunarity  (Λ)  mean±SD | 0.38±0.04 | 0.35±0.03 | 0.35±0.03 | 0.49±0.10 | 0.40±0.04 | 0.40±0.05 | 0.049* |

| Circular skeletonized ROI | | | | | | | | | |
| --- | --- | --- | --- | --- | --- | --- | --- | --- | --- |
| Region | Macular | | | | Optic disc | | | |  |
| Diagnosis | DR | G | H | DR | | G | H | p- value | |
| Box counting  dimension (Db)  mean±SD | 1.32±0.05 | 1.38±0.03 | 1.37±0.04 | 1.43±0.05 | | 1.42±0.02 | 1.40± 0.03 | <0.001* | |
| Lacunarity  (Λ)  mean±SD | 0.41±0.04 | 0.40±0.04 | 0.39±0.03 | 0.56±0.11 | | 0.47±0.04 | 0.47±0.05 | 0.055 | |

**Supplementary table 2:** Comparison between rectangular and circular region of interest (ROI) results of box counting and fractal analysis. *P- values < 0.05 are considered significant.


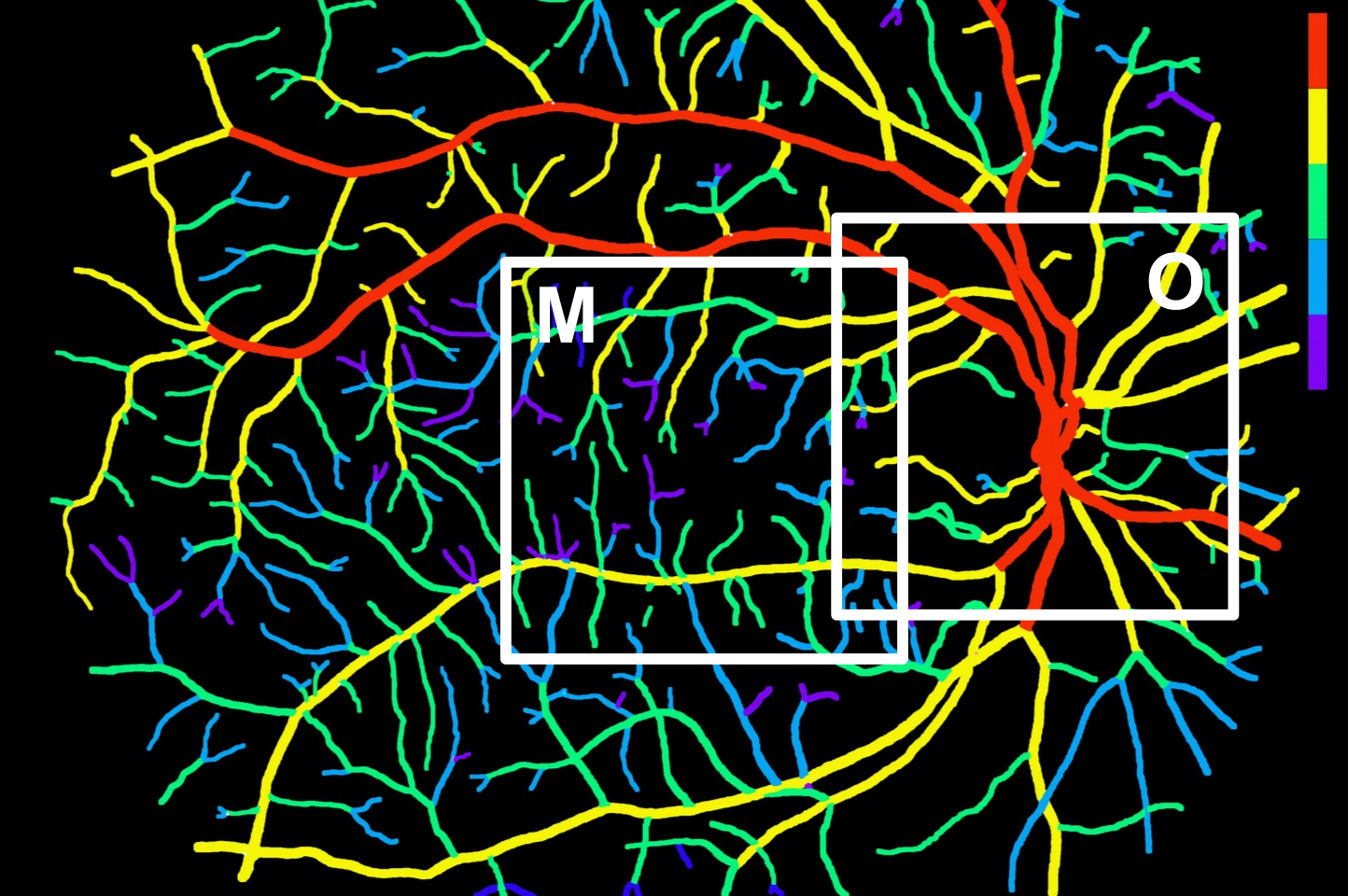


**Supplementary figure 1:** Diagram showing the two distinct regions of retina analyzed in the study. The image was captured by Canon CR-1 digital fundus camera with a 45º field of view and resolution 3504X2336 pixels and subsequently manually segmented^13^. The segmented image of the retinal microvasculature was color-coded according to branching generation, which was determined by following the method described by described by Vickerman et al^8^: red - 1^st^, yellow - 2^nd^, green - 3^rd^, blue - 4^th^, purple - 5^th^ generation of branching. The two regions of interest are labelled with white squares: M - macular region includes mostly smaller arterioles belonging to the higher number of branching generations, O - optic disc region contains small arteries and mostly larger arterioles belonging to the lower number of branching generations.

**
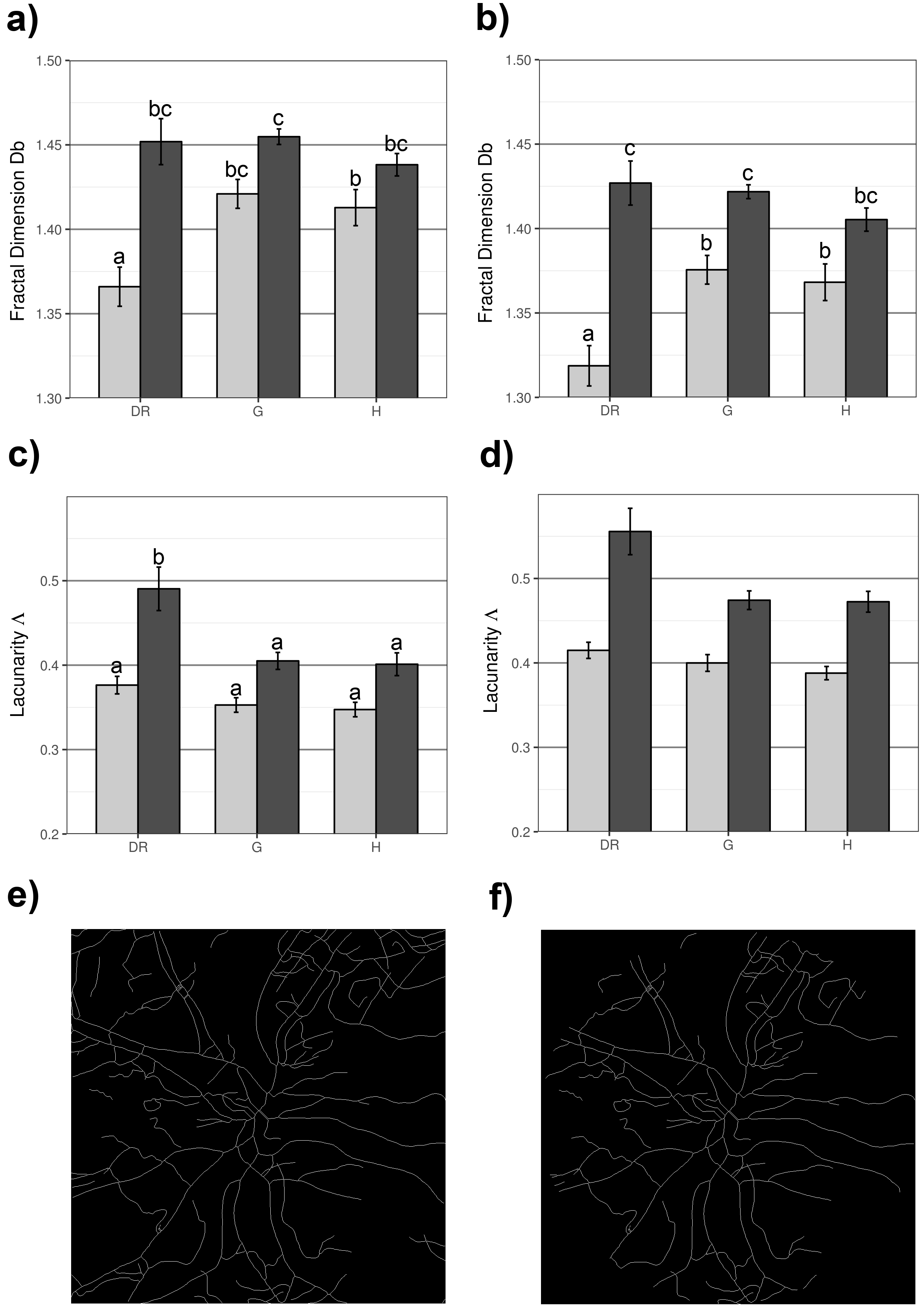
**

**Supplementary figure 2:** Comparison between rectangular and circular region of interest (ROI) results of box counting and fractal analysis. Fractal dimension (Db): A) rectangular ROI, B) circular ROI. Lacunarity Λ: C) rectangular ROI, D) circular ROI. E) Typical image of rectangular ROI, F) Typical image of circular ROI with spurious black foreground areas in all 4 corners. The groups trends observed for the rectangular and for the circular ROIs are the same. For the rectangular ROIs the absolute values for Db are generally higher and Λ values are generally lower when compared to the circular ROIs.
